# Longitudinal quality of life measurement in colorectal cancer survivorship: a scoping review of patient-reported outcome measures (PROMs)

**DOI:** 10.1007/s11764-026-02021-5

**Published:** 2026-04-22

**Authors:** Ivon Lemus Solano, Julia G. Davies, Natalie Bradford, Jessica Cameron, Kou Kou, Nirmali Sivapragasam, Susanna Cramb

**Affiliations:** 1 Australian Centre for Health Services Innovation & Centre for Healthcare Transformation, Queensland University of Technology, 60 Musk Avenue, Kelvin Grove Brisbane, QLD 4059 Australia; 2 School of Public Health and Social Work, Faculty of Health, Queensland University of Technology, Brisbane, Australia; 3 Viertel Cancer Research Centre, Cancer Council Queensland, Fortitude Valley, Australia; 4 School of Exercise and Nutrition Science, Faculty of Health, Queensland University of Technology, Brisbane, Australia; 5 Cancer and Palliative Care Outcomes Centre, Centre for Healthcare Transformation, Queensland University of Technology, Brisbane, Australia; 6 Centre for Children’s Health Research, Queensland University of Technology, Brisbane, Australia; 7 Centre for Data Science, Queensland University of Technology, Brisbane, Australia

**Keywords:** Patient-reported outcome measures, Health-related quality of life, Colorectal cancer, Survivorship

## Abstract

**Purpose:**

This study aimed to identify the Patient-Reported Outcome Measures (PROMs) used to assess health-related quality of life (HRQoL) in colorectal cancer (CRC) survivors over time, examining the specific HRQoL domains assessed by PROMs, and exploring PROM use across interventions, age groups, settings, and survivorship phases.

**Methods:**

PubMed, Embase, CINAHL, and PsycINFO were systematically searched for studies published between January 2005 and March 2025. Eligible studies focused on survivors with local or locally advanced CRC, where HRQoL was assessed at least at two timepoints (< 2 years and 5 + years post-diagnosis) using either a comprehensive PROM or two or more PROMs covering different domains and reported outcomes. Studies were screened independently by two reviewers.

**Results:**

The review included 21 articles representing 15 unique studies. Disease-specific PROMs predominated, commonly supplemented with generic instruments. Measurement timing and domain coverage varied widely. Physical health and symptom domains such as bowel function, fatigue, and pain were consistently assessed, whereas psychosocial constructs such as fear of cancer recurrence, financial strain, and social reintegration were less frequently captured. Temporal mapping of PROMs revealed strong emphasis on acute recovery within 2 years and limited long-term monitoring beyond 5 years.

**Conclusions:**

PROM use in CRC survivorship research remains fragmented. Harmonisation of measures, survivor involvement in PROM development, and longitudinal implementation are essential next steps to generate comparable, patient-centred, and policy-relevant evidence that supports long-term survivorship care.

**Scoping review registration:**

This protocol has been registered prospectively with the Open Science Framework (OSF) https://osf.io/a9rf7.

**Implications for cancer survivors:**

More consistent and long-term use of PROMs in follow-up care can help identify late treatment effects and under-recognised survivorship issues.

**Supplementary Information:**

The online version contains supplementary material available at https://doi.org/10.1007/s11764-026-02021-5.

## Introduction

Colorectal cancer (CRC) is one of the most commonly diagnosed malignancies worldwide, with more than 1.9 million new cases and over 930,000 deaths annually [1]. Due to advances in prevention, screening, and treatment, CRC survivorship has improved significantly over recent decades. In Australia, 5-year relative survival now exceeds 70% with an increasing proportion of individuals who live beyond a decade after diagnosis [2]. As CRC transitions to a chronic disease for many patients, it is increasingly important to understand patients’ long-term health-related quality of life (HRQoL), encompassing the physical, psychological, and social dimensions of wellbeing in survivorship.

Patient-reported outcome measures (PROMs) are central to this evaluation, as they capture survivors’ perspectives on functioning and well-being that are not fully evident through clinical or biomarker endpoints [3]. By systematically documenting outcomes such as fatigue, bowel function, sexual health, and emotional well-being, PROMs provide a patient-centred lens for assessing treatment impact, informing shared decision-making, and guiding survivorship care planning [4]. Moreover, aggregated PROM data supports health-system performance evaluation and quality improvement, thus reinforcing their value across the cancer care continuum [5]. Longitudinal collection of PROMs also enables early detection of late effects and evolving needs, identifying priorities for supportive or rehabilitative care [6], while enhancing communication between patients and clinicians [7]. Ultimately, PROMs help shift the focus of CRC survivorship from how long patients live to how well they live, reflecting a broader and more meaningful measure of recovery and well-being.

Although several studies have assessed HRQoL longitudinally in CRC survivors using PROMs, existing evidence has largely focused on describing HRQoL trajectories within individual cohorts [8, 9] rather than examining how HRQoL measurement itself varies over time. To our knowledge, no CRC-focused synthesis has systematically evaluated whether PROM domain selection differs across survivorship phases (baseline, short-term recovery, and long-term survivorship), using a structured, phase-specific mapping of instruments and domains, nor examined how longitudinal measurement design decisions such as assessment frequency, selective subscale reporting, or switching instruments over time may affect interpretability and comparability across studies and across populations and settings. As a result, it remains unclear whether current PROM use adequately captures phase-specific survivorship priorities or supports coherent, patient-centred longitudinal assessment across the survivorship trajectory.

This scoping review is aimed at systematically synthesising how HRQoL has been measured in CRC survivorship research. Specifically, it seeks to identify PROM use, the HRQoL domains they assess, and how these instruments are applied across survivorship phases and populations over time.

The review addresses the following four research questions:

1. What PROMs are used to measure HRQoL in CRC survivors over time?
2. What specific domains of HRQoL do these PROMs assess?
3. How are PROMs used to compare HRQoL outcomes among CRC survivors across different intervention groups, age cohorts, and geographic settings?
4. How do these studies assess changes across different timepoints within the survivorship trajectory?

## Methods

This scoping review was conducted following the methodological framework proposed by Arksey and O’Malley [10] and the recommendations by Levac et al. [11]. The Preferred Reporting Items for Systematic Reviews and Meta-Analyses extension for Scoping Reviews (PRISMA-ScR) [12] and the Joanna Briggs Institute (JBI) scoping review methodology guided the reporting [13]. The protocol was prospectively registered in the Open Science Framework (OSF) and can be accessed at https://osf.io/a9rf7.

### Search strategy

We performed a comprehensive search on March 28th, 2025, in four electronic databases: PubMed, Embase, CINAHL, and PsycINFO. The search strategy combined controlled vocabulary (MeSH terms) and free-text keywords related to “quality of life”, “health-related quality of life”, “colorectal cancer”, and “patient-reported outcome measures”. The terms were iteratively refined to ensure both sensitivity and specificity. Reference lists of included studies were also manually screened to identify additional eligible articles. The search was restricted to English-language publications involving human participants, covering articles published from January 1 st, 2005, to ensure alignment with contemporary survivorship research. This timeframe reflects the introduction and broader adoption of validated CRC-specific PROMs (e.g. the European Organisation for Research and Treatment of Cancer Quality of Life Questionnaire – Colorectal Cancer Module (EORTC QLQ-CR29)) [14] and coincides with improvements in survival rates [2] and treatment standardisation [15]. The complete search strategy for each database is provided in Supplementary File 1.

### Study selection

Eligibility criteria were structured using the Population–Concept–Context (PCC) framework recommended for scoping reviews [16]. We included studies that recruited adults (aged ≥ 18 years) diagnosed with CRC and reporting on survivors with local or locally advanced disease (stages I–III). Studies exclusively involving stage IV or metastatic CRC populations were excluded, as HRQoL in this group is largely shaped by disease progression and palliative management rather than long-term survivorship [17]. Papers including mixed-stage cohorts were eligible only if results were reported separately for stages I–III.

Studies focusing on anal cancer were excluded because it represents a biologically and clinically distinct disease entity [18, 19]. Likewise, studies limited to participants with hereditary syndromes (e.g. Lynch syndrome and Familial Adenomatous Polyposis) or inflammatory bowel disease (e.g. ulcerative colitis and Crohn’s disease) were excluded unless data for CRC were presented separately, given their unique disease courses and management considerations [20] 

The concept underpinning this review was HRQoL measured with validated PROMs. To be eligible, studies had to report HRQoL using either a comprehensive PROM or at least two instruments assessing different HRQoL domains. Studies needed to assess outcomes at a minimum of two timepoints within the same cohort: one within 2 years of diagnosis/treatment and another at least 5 years post-diagnosis/post-treatment.

This criterion was applied to ensure the review captured both short-term recovery and long-term survivorship trajectories. The ≥ 5-year threshold was adopted because it is widely used in oncology survivorship research as a conventional marker of long-term survival, reflecting the period beyond acute treatment when many patients have completed primary therapy and the risk of recurrence has substantially declined [21]. This timeframe is also commonly used to distinguish long-term survivors and to evaluate persistent or late-emerging consequences of cancer and its treatment [22, 23].

The context was unrestricted by geography or setting, allowing inclusion of international research conducted in diverse healthcare systems. Eligible publications were original, peer-reviewed research articles. Reviews, editorials, commentaries, protocols, case reports, and conference abstracts were excluded.

All references identified through the search were imported into EndNote 20, and duplicates were removed. Additional duplicate detection and screening were managed in Rayyan, a web-based platform designed to facilitate the screening and selection of studies in systematic and scoping reviews [24]. Title and abstract screening were initially conducted by one reviewer (NS) and subsequently performed independently by two reviewers (ILS and JD), applying the predefined inclusion criteria. Full-text screening of potentially eligible articles was also undertaken independently by the last two reviewers. Discrepancies were resolved through discussion and, when necessary, consultation with a third reviewer (NB).

### Data extraction and synthesis

A bespoke data extraction form was designed to record the key details of each included study. Extracted variables included study characteristics (author, year of publication, country, and study design), participant attributes (cancer stage, age, sex, and treatment details), instruments used to assess HRQoL, domains and subdomains captured, timing of PROM administration relative to diagnosis and treatment, length of follow-up, and key findings. The extraction form was pilot tested to ensure consistency across variables. Data extraction was conducted by one reviewer (ILS), and consistency was verified through iterative checking and discussion with the broader review team.

To synthesise the findings, we adopted a descriptive and mapping approach consistent with JBI methodology. PROMs were systematically charted, and the domains they assessed were grouped into conceptual categories. Aggregated counts (absolute frequencies) were calculated for each domain to summarise how HRQoL was assessed across the included articles, with counts representing the number of times a given domain was measured across instruments and study timepoints. The domains captured by the EORTC QLQ-C30 and its CRC modules (QLQ-CR29) were used as the reference framework, given their high prevalence among included studies (Supplementary File 2). Domains from other instruments were mapped against this structure to enable comparability across studies and to identify areas of consistency and gaps in the existing literature. The inclusion of both domains and subdomains in the synthesis allowed capturing the granularity of HRQoL outcomes, such as bowel function, which encompassed subdomains including frequency, urgency, and incontinence. Microsoft Excel was used to manage data extraction, generate frequency counts, and produce descriptive summaries.

This scoping review did not involve quality appraisal of individual studies, in keeping with JBI guidance, as the aim was to synthesise the evidence rather than evaluate study quality. All methodological decisions, including eligibility criteria, data charting, and synthesis procedures, were prespecified in the protocol [25]. As the review involved the synthesis of published literature, ethical approval was not required.

## Results

**Fig. 1:**
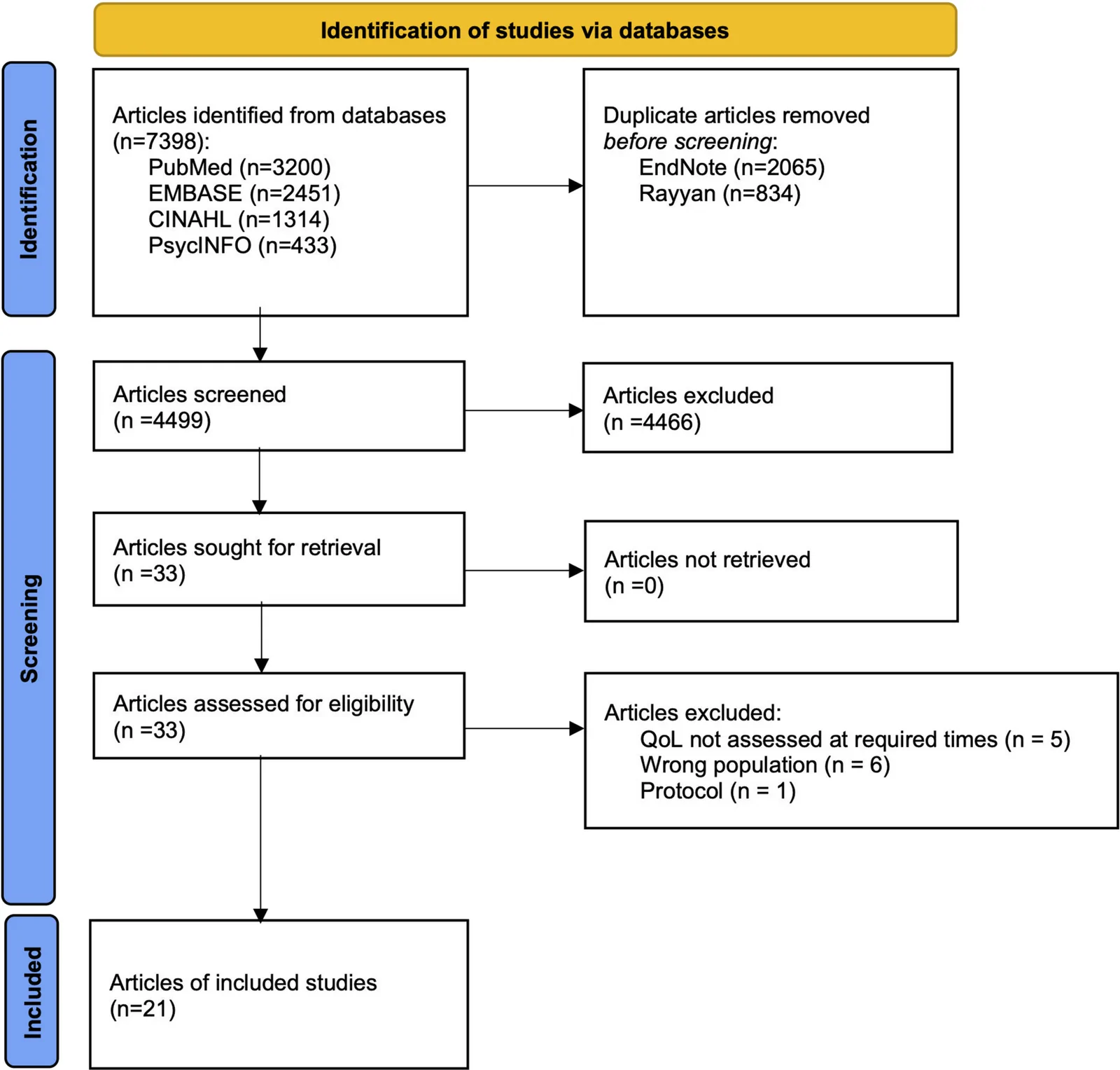
PRISMA flow diagram

A total of 7398 articles were identified from the database search. After removing duplicates using EndNote and Rayyan, 4499 unique articles remained for title and abstract screening. Subsequently, 33 articles were shortlisted for full-text screening, of which 21 articles representing 15 studies were finally included in the analysis. The article selection process is summarised in the PRISMA flow diagram [26] (Fig. 1).

### Study characteristics

The same cohorts were described in multiple articles, including a United Kingdom (UK) cohort for four articles [27–30], a Spanish cohort for two articles [31, 32], a Dutch cohort for two articles [33, 34] and an Australian cohort for two articles [35, 36].

Based on the 15 unique studies, the largest contributions were from Australia (*n* = 3, 20%), followed by the United States (USA) (*n* = 2, 12.5%), the UK (*n* = 2, 12.5%), and the Netherlands (*n* = 2, 12.5%). Single studies were conducted in Denmark, Spain, Italy, and Lithuania (each *n* = 1, 6.25%). In addition, two studies (12.5%) reported recruitment across multiple international sites: one across Norway and Sweden [37] and another across Italy, Spain, and the USA [38] (Supplementary File 3).

Prior to 2012, only one article met the inclusion criteria, and this was published in 2005. From 2012, there have been, on average, two articles per year, with a peak of six articles in 2022 (Supplementary File 4).

A variety of study designs were identified. The most frequent were prospective longitudinal cohorts (*n* = 14, representing 9 unique cohorts). Although 14 articles used this design, four reported on the same UK cohort study, “ColoREctal Wellbeing” (CREW) [27–30], two on the same Spanish cohort [31, 32] and two on the same Australian cohort [35, 36].

Randomised controlled trials (RCTs) were also identified (*n* = 6) [33, 34, 37–40], including Phase III [37, 40] and one pragmatic multicentre design [21]. Two of these RCTs were based on the same Dutch TME trial, one reporting long-term longitudinal HRQoL follow-up and another as a secondary analysis [33, 34]. Additional designs included a single-arm Phase II trial (*n* = 1) [41].

Sample sizes ranged from 40 participants [41] to 2531 in a large Spanish multicentre cohort study [31]. Participants were predominantly older adults, with reported mean or median ages ranging from 58 to 67 years across studies. A slightly larger proportion of men were included, ranging from 52 to 80% across studies (mean = 62%). The majority of articles focused on CRC (*n* = 13, 62%), although some specified colon cancer and rectal cancer separately.

The timeframe of HRQoL assessments ranged from pre-treatment phase (e.g. before surgery or initiation of therapy) to long-term follow-up, extending up to 14 years after diagnosis/treatment in the Dutch trial (TME) [34]. Most articles began measuring HRQoL before treatment commenced (*n* = 16, 76%), allowing for robust tracking of trajectories from diagnosis through follow-up. The remaining articles conducted PROM assessments shortly after treatment, within the first 1–6 months (*n* = 5, 24%). PROMs were employed to measure HRQoL in diverse contexts, and the articles spanned heterogeneous populations, geographic settings, and follow-up duration (Table 1).

**Table 1:** Article characteristics

Author	Year	Country	Study design	Sample size	Cancer subtype	No. of follow-ups	Follow-up timepoints	Type of PROM used	PROMs used
Alahmadi	2021	Australia	Prospective cohort study	710	RC	9	Baseline; 6, 12, 18, 24, and 30 months; 3, 4, and 5 years	Generic + cancer-specific	SF-36; FACT-C
Brændengen	2012	Norway, Sweden	RCT	207	RC	2	Baseline and follow-up (median 6 years)	Cancer-specific	EORTC QLQ-C30
Chambers	2012	Australia	Prospective cohort study^a^	1966	CRC	2	Baseline, 5 years	Generic + cancer-specific	FACT-C; SWLS; BSI
Charlton	2015	USA	Prospective cohort study	239	RC	3	Baseline, 1 and 7 years	Generic + non-standardised questionnaire	EQ-5D; SF-12; CanCORS Defecation Scale
Chau	2005	UK	RCT	801	CRC	20/25*	Baseline Weekly at weeks 1, 2, 3, 4 Biweekly at weeks 6, 8, 10, 12, 14, 16, 18, 20, 22, 24 1 month post-treatment (week 28) Year 1: every 3 months Year 2: every 6 months Years 3–5: annual	Cancer-specific	EORTC QLQ-C30
Chong	2024	The Netherlands	Prospective cohort study	459	CRC	6	Baseline; 6 weeks; 6 months; 1, 2, and 5 years	Cancer-specific	EORTC QLQ-C30; CIS
Cummings	2018	UK	Prospective cohort study ^b^	861	CRC	8	Baseline; 3, 9, and 15 months; 2, 3, 4, and 5 years	Cancer-specific	EORTC QLQ-C30; CR29
Cummings	2022	UK	Prospective cohort study ^b^	501	CRC	8	Baseline; 3, 9, and 15 months; 2, 3, 4, and 5 years	Generic	QLACS-GSS; EQ-5D
Dizdarevic	2020	Denmark	Other trial design	40	RC	6	Baseline; 6 months; 1, 2, 4, and 5 years	Cancer-specific	EORTC QLQ-CR29
Dunn	2013	Australia	Prospective cohort study ^a^	1966	CRC	6	5 months; 1, 2, 3, 4, and 5 years	Generic + cancer-specific	FACT-C; SWLS
Frankland	2022	UK	Prospective cohort study ^b^	790	CRC	8	Baseline; 3, 9, and 15 months; 2, 3, 4, and 5 years	Generic + cancer-specific	EORTC QLQ-C30; CR29; QLACS ^1^
Ganz	2022	USA	RCT	1608	RC	4	Baseline, post-neoadjuvant chemoradiotherapy, 1 and 5 years	Generic + cancer-specific	FACT TOI; SF-36^2^
Orive	2022	Spain	Prospective cohort study^c^	2531	CRC	5	Baseline; 1, 2, 3, and 5 years	Generic + cancer-specific	EQ-5D-5L; EORTC-QLQ-C30; HADS
Orive	2023	Spain	Prospective cohort study^c^	2531	CRC	5	Baseline; 1, 2, 3, and 5 years	Generic + cancer-specific	EQ-5D-5L; EORTC-QLQ-C30; HADS
Perrone	2023	Italy	Prospective cohort study	64	CRC	4	Baseline, end of neoadjuvant treatment, 1 year and 5 years	Cancer-specific + non-standardised questionnaire	EORTC QLQ-C30; CR38; HADS; non-standardised questionnaire
Rosati	2016	Italy, Spain, USA	RCT	1228	CRC	6	Baseline; 1, 2, 3, 4, and 5 years	Generic	SF-12, PGWB
Steffens	2024	Australia	Prospective cohort study	565	RC	11	Baseline; 6, 12, 18, 24, 30, 36, and 42 months; 5 years	Generic + cancer-specific	SF-36; FACT-C
Valeikaite-Tauginiene	2022	Lithuania	Prospective cohort study	88	CRC	4	Baseline; 1, 2, and 6 years	Cancer-specific	EORTC QLQ-C30; CR29
van Kooten	2022	The Netherlands	RCT ^d^	1207	RC	7	Baseline; 3, 6, 12, and 18 months; 2 and 14.4 years	Cancer-specific	RSCL; EORTC QLQ-C30; CR29
Wheelwright	2020	UK	Prospective cohort study ^b^	1017	CRC	8	Baseline; 3, 9, and 15 months; 2, 3, 4, and 5 years	Generic + cancer-specific	QLACS; EORTC QLQ-C30; CR29^3^
Wiltink	2016	The Netherlands	RCT ^d^	1530	RC	8	Baseline; 3, 6, 12, and 18 months; 2, 5, and 14 years	Cancer-specific + non-standardised questionnaire	RSCL; Non-standardised questionnaire; EORTC QLQ-C30; CR29^4^

*RCT* randomised control trial, *RC* rectal cancer, *CRC* colorectal cancer, *SF-36* 36-Item Short Form Health Survey, *SF-12* 12-Item Short Form Health Survey, *EQ-5D* EuroQol 5-Dimension, *EQ-5D-5L* EuroQol 5-Dimension 5-Level, *QLACS* Quality of Life in Adult Cancer Survivors Scale (generic summary score [GSS] and cancer-specific summary score [CSS]), *FACT-C* Functional Assessment of Cancer Therapy – Colorectal, *FACT-TOI* Functional Assessment of Cancer Therapy – Trial Outcome Index, *EORTC QLQ-C30* European Organisation for Research and Treatment of Cancer Quality of Life Questionnaire – Core 30, *EORTC QLQ-CR29* European Organisation for Research and Treatment of Cancer Quality of Life Questionnaire – Colorectal Cancer Module, *EORTC QLQ-CR38* European Organisation for Research and Treatment of Cancer Quality of Life Questionnaire – Colorectal Cancer Module 38, *RSCL* Rotterdam Symptom Checklist, *SWLS* Satisfaction with Life Scale, *BSI* Brief Symptom Inventory, *CIS* Checklist Individual Strength, *SEMCD* Self-efficacy for Managing Chronic Disease Scale, *CSSES* Cancer Survivor Self-Efficacy Scale, *CES-D* Center for Epidemiologic Studies Depression Scale, *STAI* State-Trait Anxiety Inventory, *MOS-SSS* Medical Outcomes Study Social Support Survey, *SCL-17* Symptom Checklist–17, *FACT-NTX13* Functional Assessment of Cancer Therapy – Neurotoxicity 13-item Version, *FACT-NTX4* Functional Assessment of Cancer Therapy – Neurotoxicity 4-item Version, *HADS* Hospital Anxiety and Depression Scale, *PGWB Index* Psychological General Well-Being Index, *PANAS* Positive and Negative Affect Schedule, *PWI-A* Personal Wellbeing Index – Adult, *IPQ-R* Illness Perception Questionnaire – Revised, *COPE* Coping Orientations to Problems Experienced, *LTE* List of Threatening Experiences, *EORTC QLQ-PR29* European Organisation for Research and Treatment of Cancer Quality of Life Questionnaire – Prostate Cancer Module, *EORTC QLQ-CX24* European Organisation for Research and Treatment of Cancer Quality of Life Questionnaire – Cervical Cancer Module, *LARS* Low Anterior Resection Syndrome Score

*Follow-up frequency in this study varied by treatment schedules, ranging from 20 to 25 PROM assessments overall. In the outlined regimen, bolus arm, QoL measurements were conducted at 25 timepoints, whereas, in the other infusion arm, assessments were performed at 20 timepoints. The difference reflects a lower number of measurements during the treatment period

^a^Australian Cohort

^b^CREW study

^c^Spanish Cohort

^d^TME Trial

^1^Additional PROMs reported in the study include QLACS (Sexual Domain); CSSES; SEMCD; CES-D; STAI; MOS-SSS

^2^Additional PROMs reported in the study include SF-36 (Vitality scale); SCL-17; FACT-NTX13; FACT-NTX4; non-standardised questionnaire; SF-36 component scores: Physical Component Summary (PCS), Mental Component Summary (MCS), and Vitality at 5 years

^3^Additional PROMs reported in the study include EQ-5D; PANAS; CES-D; STAI; MOS-SSS; CSSES; SEMCD; PWI-A; IPQ-R; COPE; LTE

^4^Additional PROMs reported in the study include EORTC QLQ-PR29; EORTC QLQ-CX24; LARS score; non-standardised questionnaire

### PROMs used to assess HRQoL

**Fig. 2:**
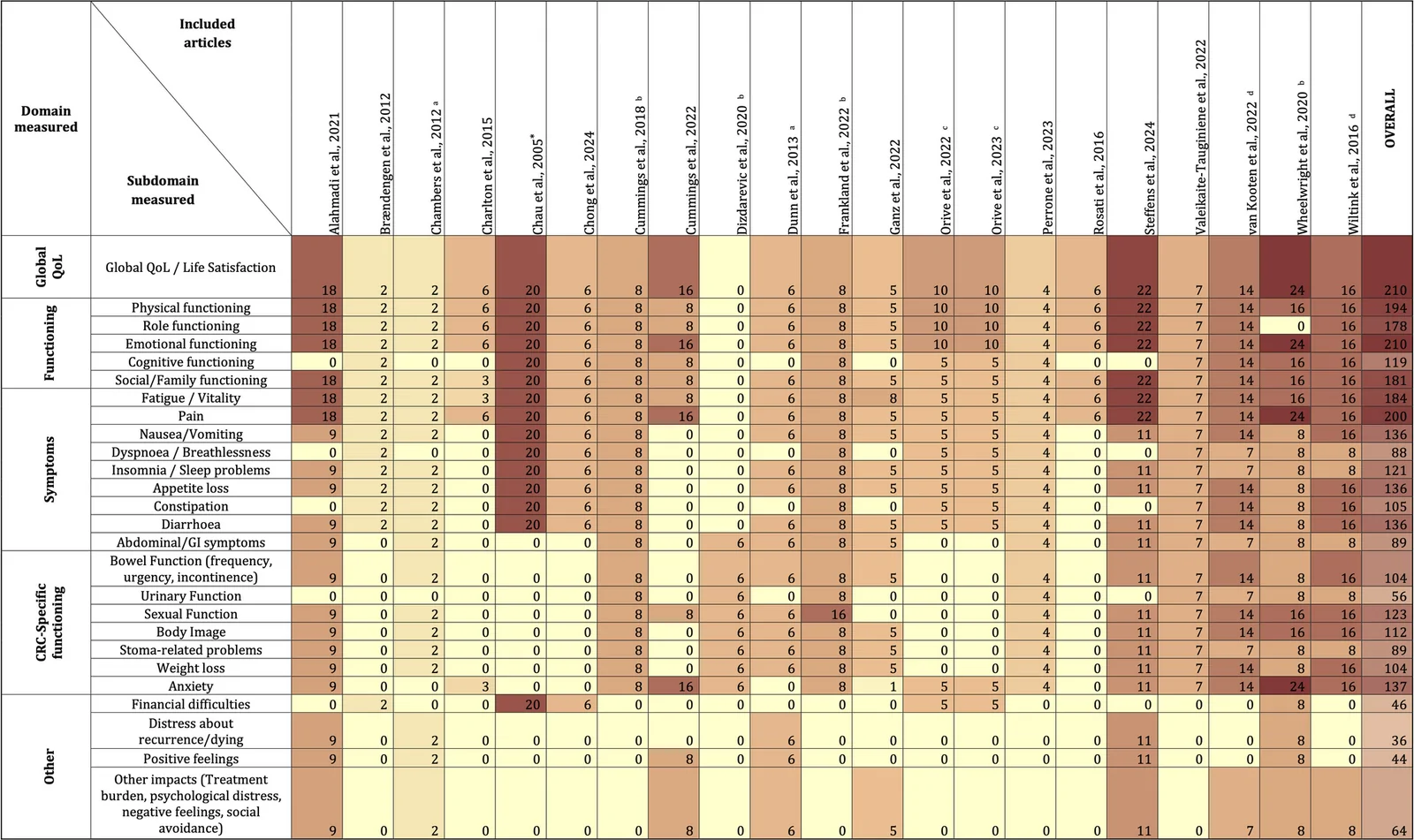
Aggregated frequencies for each included article. ^a^Australian Cohort; ^b^CREW study; ^c^Spanish Cohort; ^d^TME Trial. ^*^This study included two distinct treatment arms with different HRQoL measurement timepoints using PROMs. The overall frequencies shown in the figure are based on 20 timepoints from one arm, while the other arm, in which HRQoL was measured at 25 timepoints, is not displayed in the figure. Colour gradient indicates how frequently each HRQoL domain was assessed across studies, with darker red shades representing higher counts

Across the 21 included articles, a diverse range of PROMs were employed to assess HRQoL among CRC survivors (Fig. 2). The most commonly used instruments and their key characteristics are summarised in Table 2.

**Table 2:** HRQoL instruments table

**#**	Abbreviation used in paper	Full instrument name	Type	Brief description	Key aspects covered	Key validation paper(s)	Strengths	Shortcomings/considerations
1	EORTC QLQ-C30	European Organisation for Research and Treatment of Cancer Quality of Life Questionnaire-Core 30	Cancer-generic core questionnaire	30-item core cancer HRQoL questionnaire used across oncology populations. It has been designed to be used alone or with tumour-specific modules.	Global health/QoL; physical, role, emotional, cognitive and social functioning; fatigue, pain, nausea/vomiting; dyspnoea, insomnia, appetite loss, constipation, diarrhoea; financial difficulties.	Aaronson et al., J Natl Cancer Inst. 1993;85:365–376. https://doi.org/10.1093/jnci/85.5.365 | Osoba et al., Qual Life Res. 1997;6:103–108. https://doi.org/10.1023/A:1026429831234	Very widely used; strong validation; broad cancer-core coverage; allows comparison across cancer populations.	Not CRC-specific when used alone; limited detail on bowel, stoma, urinary and sexual outcomes; can miss survivorship issues such as fear of recurrence.
2	EORTC QLQ-CR29	EORTC Quality of Life Questionnaire-Colorectal Cancer Module (29 items)	CRC-specific module (to be used with QLQ-C30)	Updated CRC-specific companion module to the QLQ-C30, designed to capture CRC-specific concerns.	Bowel frequency, stool/urinary symptoms, bloating, body image, anxiety, weight, sexual interest/function, stoma-related problems.	Whistance et al., Eur J Cancer. 2009;45:3017–3026. https://doi.org/10.1016/j.ejca.2009.08.014	Improves sensitivity to CRC-specific symptoms and treatment effects.	Should not be treated as a stand-alone global QoL instrument; some domains are single items; still limited on broader survivorship issues such as financial toxicity or social reintegration.
3	EORTC QLQ-CR38	EORTC Quality of Life Questionnaire-Colorectal Cancer Module (38 items)	CRC-specific module largely replaced by the CR29	Predecessor to the QLQ-CR29; developed to extend the QLQ-C30 for CRC-specific symptoms and treatment side effects.	Body image, sexuality, future perspective, micturition, gastrointestinal symptoms, defaecation problems, stoma-related issues, treatment side effects.	Sprangers et al., Eur J Cancer. 1999;35:238–247. https://doi.org/10.1016/S0959-8049(98)00357-8	Important for historical comparability with older CRC cohorts and trials; allowed earlier CRC-specific assessment.	Older instrument; less aligned with contemporary survivorship issues.
4	FACT-C	Functional Assessment of Cancer Therapy-Colorectal	CRC-specific questionnaire based on FACT-G	CRC-specific extension of the FACT system combining general cancer HRQoL and colorectal-specific concerns; often used to generate total score and Trial Outcome Index (TOI).	Physical, social/family, emotional and functional well-being plus CRC-specific concerns such as bowel/stoma problems, appetite and treatment burden.	Ward et al., Qual Life Res. 1999;8:181–195. https://doi.org/10.1023/A:1008821826499	Clinically intuitive framework with strong psychometric properties; provides useful overall scores (including the TOI) and is often easier for clinicians to interpret than multiple separate symptom scales.	Less detailed than EORTC CRC modules for some bowel, urinary and sexual symptoms.
5	SF-36	36-Item Short Form Health Survey	Generic health status/HRQOL measure	Generic 36-item health-status measure widely used across diseases and populations.	Physical functioning, role limitations, pain, general health, vitality, social functioning, emotional wellbeing, mental health.	Ware & Sherbourne, Med Care. 1992;30:473–483. https://doi.org/10.1097/00005650-199206000-00002	Allows cross-disease and population comparisons; broad functional profile.	Longer than SF-12/EQ-5D; not cancer or CRC-specific; limited sensitivity to CRC-specific symptoms.
6	SF-12	12-Item Short Form Health Survey	Brief generic health status/HRQOL measure	12-item generic HRQOL instrument derived from the SF-36 that assesses physical and mental health through two summary scores.	Physical (PCS) and mental component summary (MCS) measures.	Ware et al., Med Care. 1996;34:220–233. https://doi.org/10.1097/00005650-199603000-00003	Feasible for large cohorts and repeated follow-up; allows broad physical/mental health comparison.	Less detail than SF-36; may be less responsive to subtle CRC-specific longitudinal change.
7	EQ-5D	EuroQol 5-Dimension questionnaire	Generic preference-based utility measure	Very brief generic health status measure widely used for utility scoring and economic evaluation.	Mobility, self-care, usual activities, pain/discomfort, anxiety/depression, plus EQ visual analogue scale (EQ VAS).	EuroQol Group, Health Policy. 1990;16:199–208. https://doi.org/10.1016/0168-8510(90)90421-9| Brooks, Health Policy. 1996;37:53–72. https://doi.org/10.1016/0168-8510(96)00822-6	Very brief; enables utility values and cost-utility analyses; allows comparison of health outcomes across diseases and populations.	Pronounced ceiling effects in some cancer survivor samples; limited symptom detail; does not capture CRC-specific concerns.
8	EQ-5D-5L	EuroQol 5-Dimension 5-Level questionnaire	Generic preference-based utility measure (5-level version)	Updated EQ-5D with five severity levels to improve sensitivity and reduce ceiling effects compared with the 3-level version.	Mobility, self-care, usual activities, pain/discomfort, anxiety/depression, plus EQ VAS.	Herdman et al., Qual Life Res. 2011;20:1727–1736. https://doi.org/10.1007/s11136-011-9903-x	Short, utility-based, and usually more sensitive than EQ-5D-3L; useful for economic evaluation in survivorship studies.	Still not cancer-specific; limited bowel/urinary/sexual detail; should ideally be paired with a disease-specific PROM in CRC research.
9	QLACS	Quality of Life in Adult Cancer Survivors	Cancer survivorship-specific QoL measure	Instrument developed specifically for long-term cancer survivorship.	Generic domains (negative/positive feelings, cognitive problems, pain, fatigue, sexual problems, social avoidance) and cancer-specific domains (appearance concerns, financial problems, distress about recurrence, family-related distress, benefits of cancer).	Avis et al., Qual Life Res. 2005;14:1007–1023. https://doi.org/10.1007/s11136-004-2147-2| Avis et al., Health Qual Life Outcomes. 2006;4:92. https://doi.org/10.1186/1477-7525-4-92	Survivorship-relevant content beyond symptoms; useful for long-term follow-up; captures recurrence worry and social/appearance issues better than many acute oncology tools.	Longer instrument; not CRC-specific; less established than EORTC/FACT in active-treatment oncology trials.
10	RSCL	Rotterdam Symptom Checklist	Cancer symptom/distress checklist	Cancer-focused symptom and distress instrument developed for repeated monitoring in clinical research.	Physical symptom distress, psychological distress, and activity level/function.	de Haes et al., Br J Cancer. 1990;62:1034–1038. https://doi.org/10.1038/bjc.1990.434	Useful for repeated symptom monitoring; sensitive to physical and psychological distress during treatment and recovery.	Older and less commonly used today; narrower survivorship coverage than modern multidomain PROMs; not CRC-specific.

Cancer-specific instruments predominated, particularly the EORTC QLQ-C30, which was reported in 12 articles [28–34, 37, 39, 42–44]. Among these, seven articles combined the EORTC QLQ-C30 with a CRC-specific module, either the QLQ-CR29 or its older version, the European Organisation for Research and Treatment of Cancer Quality of Life Questionnaire – Colorectal Cancer Module 38 (EORTC QLQ-CR38) [28–30, 33, 34, 43, 44] to capture disease-specific domains such as bowel, urinary, and sexual functioning, while four studies (five articles) used the EORTC QLQ-C30 alone [31, 32, 37, 39, 42]. One article assessed the EORTC QLQ-CR29 as an individual instrument [41]. Also, the Functional Assessment of Cancer Therapy – Colorectal (FACT-C) was used in five articles [35, 36, 40, 45, 46], including one that specifically analysed the FACT - Trial Outcome Index subset (FACT-TOI) [40].

Generic HRQoL measures were also common, with the 36-Item Short Form Health Survey (SF-36) applied in three studies [40, 45, 46] (one focusing exclusively on the vitality domain [40]) and the 12-Item Short Form Health Survey (SF-12) used in two studies [38, 47]. In addition, five articles (four studies) used generic utility measures that provide a single summary score, including the EuroQol 5-Dimension (EQ-5D) [27, 30, 47] and the Euro QoL 5-Dimension 5-Level (EQ-5D-5L) [31, 32].

Less commonly used instruments included the Quality of Life in Adult Cancer Survivors (QLACS), which was applied in three articles (one study) with distinct scopes: one analysed both the Generic Summary Score and Cancer-Specific Subscales components together [30], one centred on the Generic Summary Score only [27], and another focused solely on the sexual domain [29]. Additionally, the Rotterdam Symptom Checklist (RSCL) was used in the Dutch TME trial [33] [34], which at long-term follow-up also incorporated broader symptoms and functional domains, including bowel, urinary, and sexual function, captured through additional questionnaires beyond the core HRQoL measures [34].

### Domains of HRQoL assessed by PROMs

**Fig. 3:**
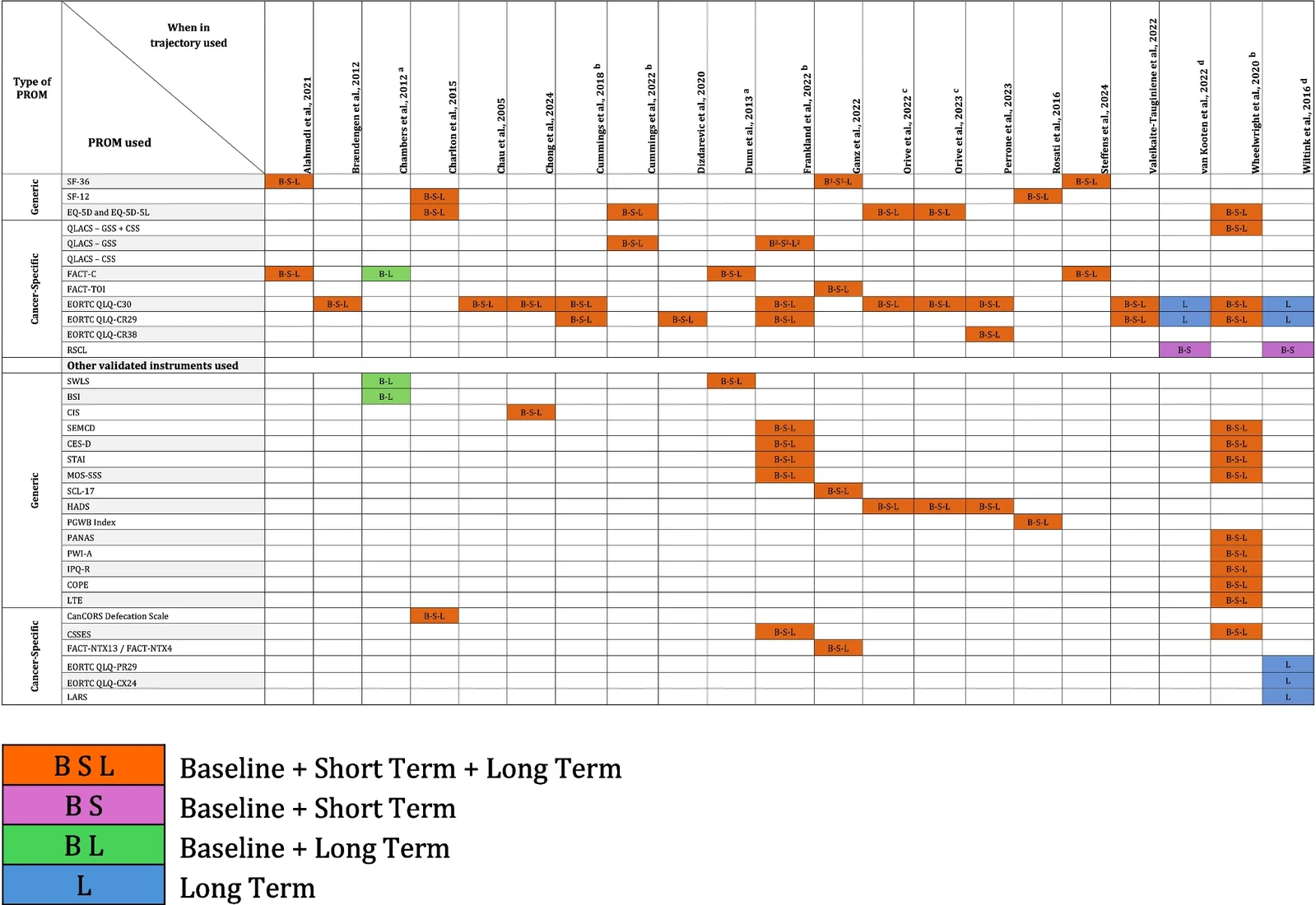
Timing of PROM administration in survivorship. Colour coding indicates the survivorship phases in which each PROM was administered across studies: Orange = Baseline + Short Term + Long Term; Purple = Baseline + Short Term; Green = Baseline + Long Term; Blue = Long Term. Abbreviations: B = Baseline; S = Short-term; L = Long-term; SF-36 = 36-Item Short Form Health Survey; SF-12 = 12-Item Short Form Health Survey; EQ-5D = EuroQol 5-Dimension; EQ-5D-5L = EuroQol 5-Dimension 5-Level; QLACS = Quality of Life in Adult Cancer Survivors Scale (generic summary score [GSS] and cancer-specific summary score [CSS]); FACT-C = Functional Assessment of Cancer Therapy – Colorectal; FACT-TOI = Functional Assessment of Cancer Therapy – Trial Outcome Index; EORTC QLQ-C30 = European Organisation for Research and Treatment of Cancer Quality of Life Questionnaire – Core 30; EORTC QLQ-CR29 = European Organisation for Research and Treatment of Cancer Quality of Life Questionnaire – Colorectal Cancer Module; EORTC QLQ-CR38 = European Organisation for Research and Treatment of Cancer Quality of Life Questionnaire – Colorectal Cancer Module 38; RSCL = Rotterdam Symptom Checklist; SWLS = Satisfaction with Life Scale; BSI = Brief Symptom Inventory; CIS = Checklist Individual Strength; SEMCD = Self-efficacy for Managing Chronic Disease Scale; CSSES = Cancer Survivor Self-Efficacy Scale; CES-D = Center for Epidemiologic Studies Depression Scale; STAI = State-Trait Anxiety Inventory; MOS-SSS = Medical Outcomes Study Social Support Survey; SCL-17 = Symptom Checklist–17; FACT-NTX13 = Functional Assessment of Cancer Therapy – Neurotoxicity 13-item Version; FACT-NTX4 = Functional Assessment of Cancer Therapy – Neurotoxicity 4-item Version; HADS = Hospital Anxiety and Depression Scale; PGWB Index = Psychological General Well-Being Index; PANAS = Positive and Negative Affect Schedule; PWI-A = Personal Wellbeing Index – Adult; IPQ-R = Illness Perception Questionnaire – Revised; COPE = Coping Orientations to Problems Experienced; LTE = List of Threatening Experiences; EORTC QLQ-PR29 = European Organisation for Research and Treatment of Cancer Quality of Life Questionnaire – Prostate Cancer Module; EORTC QLQ-CX24 = European Organisation for Research and Treatment of Cancer Quality of Life Questionnaire – Cervical Cancer Module; LARS = Low Anterior Resection Syndrome Score. ^a^Australian Cohort; ^b^CREW study; ^c^Spanish Cohort; ^d^TME Trial. ^1^At baseline and short-term follow-up, only the SF-36 Vitality subscale was administered. The full SF-36, including Physical Component Summary (PCS), Mental Component Summary (MCS), and Vitality, was assessed at long-term. ^2^Only the sexual domain of the QLACS was administ

Assessment schedules varied substantially across articles. For instance, some studies assessed domains only twice in the short term, whereas others implemented more intensive schedules with multiple short-term assessments. Nevertheless, certain areas consistently emerged as prioritised (Fig. 3).

Global QoL and emotional functioning were included in the most frequently assessed domains (*n* = 210 each), underscoring their central role in survivorship research. Pain was also widely measured (*n* = 200), followed by physical functioning (*n* = 194), fatigue/vitality (*n* = 184), and social/family functioning (*n* = 181). Role functioning remained a recurrent focus (*n* = 178), whereas cognitive functioning was less frequently assessed (*n* = 119).

Psychological and CRC symptom-specific domains were also commonly addressed. Anxiety (*n* = 137) was most frequently assessed, followed closely by diarrhoea, appetite loss, and nausea/vomiting (*n* = 136 each). Sexual function (*n* = 123), insomnia or sleep problems (*n* = 121), and body image (*n* = 112) were less consistently measured but remain central concerns, reflecting the enduring impact of treatment-related side effects. Bowel function problems (frequency, urgency, or incontinence), constipation, and weight loss were assessed at comparable levels (*n* = 104–105 each), while stoma-related issues, abdominal/GI symptoms, and breathlessness (*n* = 89, 89, 88 each) were also captured at similar levels, further highlighting functional limitations with implications for HRQoL.

Other symptoms were captured less often. Psychosocial and contextual domains, including treatment burden, psychological distress, negative feelings, and social avoidance, were sporadically considered (*n* = 64). Urinary function was evaluated less frequently (*n* = 56), while financial difficulties were even less common (*n* = 46), though their inclusion reflects the broader challenges faced beyond treatment. Domains reflecting positive feelings (*n* = 44) or distress about recurrence/dying were among the least frequently represented (*n* = 36).

It is important to recognise that many of the PROMs identified in this review were originally developed to assess treatment-related symptoms and patient experiences during active cancer treatment. As a result, these instruments may be less sensitive to domains that become more relevant several years after diagnosis, such as long-term functional adaptation, social reintegration, financial impacts, and survivorship identity.

### Variation in HRQoL outcomes across interventions, cohorts, and settings

Across intervention groups, PROMs such as the EORTC QLQ-C30, together with its CRC-specific modules (CR29/CR38), were commonly used to assess whether surgical or oncological approaches influenced survivors’ HRQoL [28–34, 37, 41, 42, 44]. In surgical trials, other instruments used, such as the FACT-TOI and the SF-36, enabled comparisons between procedures (e.g. sphincter-sparing versus abdominoperineal resections) by examining both global HRQoL scores and vitality-specific domains [40]. Chemotherapy studies likewise employed PROMs to capture treatment-related side effects and recovery trajectories, often contrasting delivery modes (e.g. infusion vs. bolus) [39].

Across age cohorts, PROMs were applied to explore how survivors of different ages experienced HRQoL. Instruments such as the EORTC QLQ-C30 and generic tools such as the SF-36 were used to capture age-related contrasts in global and functional domains. Some studies suggested that older survivors reported stable or even better mental HRQoL [45], while others identified poorer outcomes in those aged >80 years, particularly in physical functioning and mobility domains [27].

Across geographic settings, PROMs facilitated cross-country and regional comparisons, despite heterogeneity in measurement. In Northern Europe, national cohorts employed population-based approaches with repeated PROM assessments, including the EORTC QLQ-C30 in most cases, followed by CR29, and the RSCL, often reporting stable or improving HRQoL [30, 33, 37, 39, 41, 44]. By contrast, Spanish cohorts highlighted persistent psychosocial concerns despite gradual improvements using the EORTC QLQ-C30 and the EQ-5D-5L [31, 32], while Australian cohorts applied PROMs such as the SF-36 and the FACT-C to evaluate outcomes following extensive procedures such as pelvic exenteration [45, 46]. In the USA, the SF-36 (vitality domain), the EQ-5D, and the SF-12 were used to investigate long-term functional sequelae, including bowel dysfunction, with findings shaped by treatment modality and comorbidity [40, 47].

### Timing of PROM administration in survivorship

Most articles (*n* = 15) initiated PROM collection at baseline, before the onset of active treatment such as surgery, chemotherapy, or radiotherapy [27–34, 37, 40–46]. Capturing pre-treatment data allowed for a direct comparison of HRQoL trajectories from diagnosis through recovery and survivorship. The remaining articles (*n* = 6) began PROM assessments after treatment initiation, most often within the first 3 to 6 months [35, 36, 38, 39, 47]. These designs focused more on the recovery phase, capturing the early decline in functioning and the gradual return, or persistence of symptoms.

Follow-up schedules varied considerably (Table 1). Four articles adopted repeated assessments at 1, 2, 3, and 4 years, extending to 5 years [31, 32, 35, 38] while others (*n* = 10) conducted more intensive monitoring in the first post-treatment/intervention year and a half, with monthly or quarterly assessments [27–30, 33, 34, 39, 41, 45, 46]. These frequent early timepoints were particularly useful for documenting short-term complications, side effects, and the steep recovery trajectory observed in some survivors. By contrast, assessments beyond 2 years tended to be spaced further apart, reflecting a shift toward monitoring long-term adjustment and late-emerging survivorship issues.

Chau et al. [39] reported the most intensive follow-up schedule among included studies. PROMs were collected frequently during active treatment, followed by structured post-treatment and longitudinal follow-up extending to 5 years. Assessment intensity differed between treatment arms, resulting in 20–25 measurement timepoints overall. This design enabled detailed assessment of short-term fluctuations alongside longer-term HRQoL trajectories [39].

All included studies reported outcomes at 5 years post-diagnosis or longer, consistent with the review’s inclusion criteria. 17 articles reported data specifically at 5 years, while the remaining four extended follow-up beyond that point. Three articles continued evaluations at 6 [37], 7 [47], or even 14 years post-diagnosis [33, 34], offering rare extended perspectives. The Dutch cohort with 14.4 years of follow-up is particularly noteworthy, as it highlights the persistence of bowel and sexual dysfunction long after treatment, as well as subtle improvements in emotional functioning over time [29, 30].

Despite this consistency in long-term follow-up, methodological heterogeneity was evident (Table 1, Fig. 3). Some studies maintained the same PROM throughout the entire trajectory, enhancing comparability across timepoints. Others, however, switched instruments between baseline and later follow-ups, introducing challenges for synthesising trends. Nevertheless, the overall evidence base underscores a growing recognition of survivorship as a chronic phase of care.

## Discussion

The findings of this scoping review provide a comprehensive overview of the heterogeneous but evolving landscape of PROM use in CRC survivorship research. The integration of both short- and long-term assessments across studies reflects an increasing recognition that survivorship represents a prolonged phase of care in which the evolving and enduring impact of CRC and its treatments on survivors’ HRQoL must be captured.

Across the 21 included articles reporting on 15 unique studies, PROMs were used to capture a wide array of HRQoL domains, reflecting both the multidimensional nature of survivorship and the ongoing lack of standardisation in measurement approaches. While the growing inclusion of PROMs in CRC survivorship research is encouraging, this review highlights persistent conceptual and methodological gaps that hinder direct study comparability and limit translation into practice.

A central observation was the predominance of disease-specific instruments, particularly the EORTC QLQ-C30 with its CRC module (QLQ-CR29) and the FACT-C, which were used to capture symptom and functional domains central to CRC survivorship. These PROMs address constructs such as bowel, urinary, and sexual functioning, alongside pain, fatigue, emotional well-being, and social functioning [28–37, 39, 41–46]. These results highlight important gaps in existing measures frequently used in CRC research. While instruments such as the EORTC QLQ-C30 and its CRC module (QLQ-CR29) capture many treatment-related symptoms and functional impairments, they do not fully assess several domains that may become increasingly relevant during long-term survivorship. These include persistent treatment sequelae (e.g. neuropathy and chronic fatigue), psychological adaptation, financial toxicity, social participation, and evolving health behaviours [56, 57].

The dominance of those two instruments in our study aligns with international oncology practice, where these tools remain the most frequently validated for CRC populations [48]. However, their application across studies was not uniform. Selective subscale use and inconsistent administration schedules produced incompatible metrics despite the use of the same instrument name. This methodological dispersion has also been noted across oncology research more broadly [49] and underscores why the instrument name alone is an unreliable indicator of comparability. For instance, the Dutch TME trial alternated PROMs between baseline and long-term follow-up, compromising the interpretation of temporal trends [33, 34], while other cohorts limited assessment to a narrow domain such as vitality [40] or sexual function [29], even when there are available, specific standardised instruments for such domains.

Beyond instrument selection, variation in the timing and frequency of data collection limited comparability across studies and complicated the interpretation of longitudinal HRQoL assessment patterns. Follow-up schedules ranged from dense assessments during active treatment to sparse long-term evaluations. Although early measurement captures acute effects, long intervals risk missing fluctuations in recovery or delayed treatment sequelae. Longitudinal cohorts with extended monitoring, such as the Dutch TME study (> 14 years), demonstrated that persistent bowel dysfunction and gradual emotional recovery can only be understood through sustained assessment [34]. Despite this, most studies ended within 5 years post-treatment, reinforcing the need for survivorship research extending beyond the conventional 5-year benchmark.

Temporal mapping represented a key methodological contribution of this review. By distinguishing baseline, short-term (≤ 2 years), and long-term (≥ 5 years) phases, the review clarified how measurement emphasis evolves over time. Early follow-up predominantly focused on treatment-related toxicity, pain, and physical recovery, whereas later assessments were more likely to capture fatigue, emotional health, and social reintegration, though inconsistently. Within this temporal framework, psychological recovery emerged as one of the domains that extended across all survivorship phases, with emotional well-being improving more slowly than physical functioning [36]. These findings are consistent with Calman et al. [50], who, in a large prospective cohort of individuals with nonmetastatic CRC, found that clinically significant levels of depression persisted for up to 5 years after surgery despite overall mean scores improving over time. Aligned with our synthesis, their analysis underscored the enduring nature of psychological distress in survivorship and highlighted the importance of early screening and continued psychosocial support throughout the post-treatment trajectory. Comparable patterns were also reported in long-term European cohorts, where standardised instruments effectively captured global recovery but often failed to detect more subtle late survivorship challenges, such as social participation and employment reintegration [51], reinforcing the value of sustained, phase-specific PROM collection and the importance of harmonising timepoints across studies.

Our results align with previous research showing an imbalance between physical and psychosocial measurements. Physical, functional, and symptom-related domains dominate CRC HRQoL assessment, while psychosocial constructs, particularly fear of cancer recurrence, social connectedness, and role reintegration, remain under-measured [52, 53]. Fear of cancer recurrence, among the most distressing survivorship concerns, was not often included in CRC studies despite strong evidence from other cancers showing its long-term persistence and impact on HRQoL [7]. A recent Danish national survey of CRC survivors found high fear of recurrence prevalence and clear associations with fatigue and psychological distress [7]. Similarly, financial strain was rarely assessed, despite substantial evidence that economic burden is a major determinant of long-term well-being [5], even in countries where governments cover health care [54, 55]. These gaps emphasise a persistent misalignment between what survivors prioritise and what researchers measure.

In response to the limitations of existing HRQoL instruments for capturing long-term survivorship experiences, more recent efforts have focused on developing instruments specifically designed to capture the experiences of long-term cancer survivors. For instance, the EORTC Quality of Life Group has initiated the development of a survivorship-specific HRQoL questionnaire intended for disease-free survivors following completion of primary treatment. Early work in this programme identified a broad range of survivorship issues that extend beyond those captured in traditional oncology HRQoL instruments, including concerns related to fear of recurrence, social reintegration, return to work, long-term physical side effects, and changes in life priorities and identity [56, 57]

Survivor-driven PROM development and co-design also offer a potential solution. Emerging evidence from Denmark demonstrates the feasibility of involving survivors directly in PROM item generation, leading to instruments that capture financial toxicity, family relationships, and social role functioning [7]. Integrating survivor perspectives into PROM design and selection improves face validity, enhances implementation acceptability, and ensures that measured outcomes reflect meaningful aspects of life after cancer [58].

PROMs were also used for comparative evaluation across interventions, age groups, and geographic settings. In surgical research, they enabled meaningful comparison between different procedures, revealing persistent differences in bowel function and overall HRQoL according to the type of surgery [40, 45, 46]. Chemotherapy trials comparing infusion versus bolus regimens captured differing side-effect profiles, demonstrating PROMs’ capacity to quantify treatment burden from the survivor perspective [39]. However, instrument heterogeneity restricted synthesis across studies and reduced the potential for PROM data to inform clinical decision-making.

Variation in HRQoL measurement across age groups and regions also complicated interpretation. Some studies assessed emotional functioning more consistently among older survivors [31, 45] whereas others focused on physical functioning declines in those aged over 80 years [27]. Geographical differences were also evident in the domains selected for evaluation: Northern European studies frequently included longitudinal assessment of global and physical HRQoL [37, 41, 44], Spanish cohorts placed greater emphasis on psychosocial outcomes [31, 32], and Australian and US studies more often incorporated bowel and sexual function measures [45, 46]. These patterns may reflect differences in local healthcare systems and research priorities, which could influence which aspects of survivorship are most frequently captured.

Despite increasing policy attention to patient-centred survivorship care, no consensus has emerged yet on which domains constitute a CRC-specific Core Outcome Set [9]. The multiplicity of PROMs and domain structures complicates both evidence synthesis and clinical application. International efforts to define minimum standard outcome sets for cancer (e.g. The International Consortium for Health Outcomes) have yet to achieve consistent implementation in CRC survivorship research [59]. Adapting these frameworks to include survivor-identified priorities, such as fear of cancer recurrence, financial impact, and social participation, could substantially improve comparability and policy relevance [56, 57].

Interpretation of this review should consider several limitations common to the included literature. Most included studies were conducted in high-income regions (Western Europe, the USA, Australia), limiting generalisability to lower-resource settings where employment, family roles, and financial risk differ substantially. Similar disparities have been reported in global survivorship studies, showing that access to follow-up, social reintegration, and financial recovery are shaped by resource availability [60]. Furthermore, convenience sampling from tertiary centres may have over-represented survivors with better follow-up access, and attrition over time often biases long-term estimates toward healthier participants.

Several publications included in this study were derived from overlapping cohorts, meaning that the 21 included articles represented 15 unique studies. While this approach allowed for detailed longitudinal and content analysis within cohorts, it may limit the scope of independent evidence and introduce potential overrepresentation of certain populations or instruments.

This review also had notable strengths relative to prior evidence. It is, to our knowledge, the first to establish comparability by harmonising domains conceptually and quantifying their measurement frequency across baseline, short-term, and long-term survivorship phases. This approach revealed convergence in the domains repeatedly assessed (e.g. physical, bowel, and emotional domains), alongside systematic omissions (e.g. fear of cancer recurrence, social connectedness, financial toxicity, and employment). The framework is transferable as it can inform Core Outcome Set development and be adapted to other types of malignancies and chronic conditions.

Implications for research, practice, and policy follow directly. For researchers, the domain map provides an evidence-based foundation for PROM selection and scheduling to ensure coverage of priority outcomes at appropriate phases. For clinicians, it highlights the need for a holistic survivorship model extending beyond symptom control to include psychosocial and socioeconomic recovery. For policymakers, the growing emphasis on outcome standardisation, illustrated by initiatives such as The International Consortium for Health Outcomes’ CRC set, offers a feasible trajectory toward internationally comparable, patient-relevant outcomes [59].

In summary, this comprehensive review transformed instrument-level heterogeneity into a structured, phase-specific synthesis of CRC survivorship measurement. By mapping domains conceptually and identifying systematic gaps, it offers a pragmatic roadmap for advancing PROM research and integrating patient-centred outcomes into survivorship care. Harmonisation of measures, survivor involvement in PROM development, and longitudinal implementation are essential next steps to ensure that future research captures not only how long CRC survivors live, but how well they live.

## Supplementary Information

Below is the link to the electronic supplementary material.

ESM 1 (XLSX 23.8 KB)

ESM 2 (XLSX 2.44 MB)

ESM 3 (XLSX 11.3 KB)

ESM 4 (XLSX 14.5 KB)

## Data Availability

No datasets were generated or analysed during the current study.
